# Surgical Management of Recurrent Brain Metastases: A Review

**DOI:** 10.3390/cancers18162671

**Published:** 2026-08-18

**Authors:** James W. Sampson, Eric A. Goethe, Sherise D. Ferguson

**Affiliations:** 1 Texas A&M School of Engineering Medicine, Houston, TX 77030, USA; jamessampson@tamu.edu; 2 Department of Neurosurgery, University of Texas MD Anderson Cancer Center, Houston, TX 77030, USA; goethe@bcm.edu; 3 Department of Neurosurgery, Baylor College of Medicine, Houston, TX 77030, USA

**Keywords:** brain metastasis, recurrence, stereotactic radiosurgery, laser interstitial thermal therapy, brachytherapy

## Abstract

As survival for cancer patients improves, the incidence of brain metastases has risen. This is likely due to improved systemic disease control, increased diligence in surveillance imaging in high-risk pathologies, improved neuro-imaging techniques and increased systemic screening for clinical trial enrollment. While there are well-established treatments for brain metastases, many patients will experience recurrence after definitive treatment. The management of these recurrent lesions is not well established and often varies on a per-patient basis, owing to the clinical complexity and variety of these patients. Patients with recurrent brain metastases have surgical procedural options to achieve local tumor control, including laser interstitial thermal therapy (LITT) repeat open surgical resection with or without placement of intracavity brachytherapy. Repeat resection can offer rapid improvement in neurological symptoms, performance status, and potentially survival. LITT is less invasive than a standard craniotomy but offers a chance at directed local treatment while still obtaining tissue for diagnostic purposes and achieving acceptable survival outcomes. Further study is needed to determine the role of LITT for recurrent brain metastases, but it is a useful tool, particularly for patients with deep-seated lesions who may not tolerate a large surgery. Intracavitary brachytherapy allows for the immediate delivery of highly conformal radiation to the surgical bed with excellent local control and low rates of radiation necrosis, even in previously irradiated patients. The decision regarding which of the above to employ for recurrent brain metastases will vary on a case-by-case basis, and further studies are needed to standardize their use for this growing problem.

## Simple Summary

The management of recurrent brain metastases is not standardized, owing to patient heterogeneity and medical complexity. Many local, interventional options exist for recurrent brain metastases, including laser interstitial thermal therapy, repeat craniotomy with adjuvant therapy and/or intracavitary brachytherapy.

## 1. Introduction

As the survival of cancer patients improves with advances in treatment, the incidence of brain metastases is increasing [1]. Approximately 15% of all cancer patients will develop brain metastases, and, despite well-established treatment (surgical resection, chemotherapy, and radiation), between 40 and 60% will experience central nervous system (CNS) recurrence [1,2,3]. Most of these recurrences will happen within a year of treatment [4]. While most patients with metastatic cancer die of progressive extracranial disease, approximately 10% will die of uncontrolled brain metastasis [Scnurmann] [5]. Patients with uncontrolled CNS disease typically die from neurologic causes, so local control of brain metastases can have a significant impact on patient morbidity and mortality [1]. The treatment of newly diagnosed brain metastases is informed by well-established guidelines [6]. While there are multiple treatment options available, including reoperation, re-irradiation, or systemic treatment, the evidence guiding treatment of recurrent brain metastases is limited, and treatment decisions are typically made on an individual, tailored basis [7,8,9]. This narrative review will discuss the neurosurgical management of recurrent brain metastasis. For the purposes of this review, we define “recurrence” as brain metastases that have undergone/completed and subsequently failed first-line definitive local therapy (i.e., stereotactic radiosurgery [SRS], surgical resection).

## 2. Causes of Local Recurrence of Brain Metastasis

A high rate of recurrence warrants an understanding of associated risk factors. One of the most consistently reported risk factors is incomplete resection at initial operation [10,11,12]. Patel et al. [3] reported that larger tumor size and piecemeal (intralesional) resection were associated with increased risk of local recurrence in single brain metastases not treated with adjuvant radiation. More recent studies have also highlighted the impact of post-operative residual tumor burden on patient outcome [11,13,14,15,16,17]. Aftahy et al. reported their findings in 704 surgical patients; in this cohort, complete cytoreduction was achieved in 69% of patients. Although their focus was not local control, these authors reported that both pre-operative and post-operative tumor burden had a significant impact on patient survival [11]. In a recent study that included 867 surgical brain metastasis patients, residual tumor on post-operative MRI was correlated with both worse progression-free survival and overall survival [17].

Given microscopic patterns of tumor cell invasion into surrounding brain, lack of or a delay in receiving adjuvant systemic treatment or radiation may also increase the risk of local recurrence. O’Brien et al. retrospectively reviewed 159 patients undergoing resection and referred for adjuvant SRS and reported that 12% of patients never received adjuvant therapy. Furthermore, the authors reported a correlation between time to post-operative SRS and local recurrence. Specifically, recurrence rates were 2.3%, 14.5% and 48.5% for patients receiving adjuvant SRS at ≤4 weeks, >4–8 weeks and >8 weeks post-operatively, respectively [18]. Another study of 133 post-operative patients also noted that time to adjuvant SRS was predictive of local recurrence; time from surgery to SRS was 34 days for patients without recurrence versus 61 days for those with local recurrence, highlighting the impact of this issue [19].

Genomic alterations may also play a part in local recurrence, as analysis of next-generation sequencing data from 570 patients by Lamba et al. [20] identified several genes associated with differential risk of recurrence following whole-brain radiation therapy (WBRT) and SRS. In addition to identifying multiple key genomic alterations, the authors’ multivariable analysis noted that metastasis-associated edema, tumor size and WBRT without local therapy were all associated with local failure post radiation. For tumors treated upfront with SRS, tumor size has been associated with risk of local recurrence. In their study of 2268 patients with 6308 brain metastases, Rashid et al. reported that lesions >1 cm had significantly higher risk of local recurrence compared to lesions <0.5 cm [21]. In addition to tumor size, intrinsic tumor characteristics can also be impactful. A study of 2211 newly diagnosed brain metastases treated with upfront radiation (SRS or WBRT) noted that cystic lesions were more likely to recur following SRS compared to solid metastatic lesions. Specifically, the one-year local control rates for cystic versus solid metastases were 75% versus 88%, respectively [22].

## 3. Repeat Resection

Despite the potential oncological benefits of definitive cytoreduction, open surgical resection is only performed in 1% to 11% of patients with recurrent brain metastases (see Table 1) [23]. This low percentage may be due to patient functional status, medical complexity that may influence anesthetic risk (hypertension, diabetes, anticoagulant therapy, etc.), concomitant progression of systemic disease, inability to hold systemic therapy for a surgical procedure, limited post-operative treatment options and overall balancing of life expectancy with patient quality of life, particularly in high-risk surgical locations. For the limited number of selected patients who are candidates for re-resection, surgery appears to provide a survival benefit; this finding should be interpreted with caution; however, as patients eligible for repeat resection are likely healthier, have limited systemic disease burden, have a smaller raw number of brain metastases and have more surgically accessible lesions. Several authors have examined the role of surgical resection for recurrent brain metastases and demonstrated an association between repeat resection and improvements in survival. Even earlier reports that predate advances in systemic therapy indicate the value of repeat resection. Sundaresan et al. [24] observed neurologic improvement in 66% and median survival of 9 months after reoperation in a series of 21 patients with recurrent brain metastases. In a retrospective review of 67 patients with recurrent brain metastases, Schackert et al. [4] observed a median survival of 7.5 months following repeat resection. In this study, the authors reported that time to recurrence was correlated with overall post-operative survival. Patients with recurrence > 200 days after initial treatment had significantly better survival (9.2 months) than patients with recurrence within 200 days (6 months). Furthermore, patients with complete resection survived longer than those with residual tumor (11.1 vs. 4.8 months).

More modern series of resection of recurrent metastases are likewise favorable, particularly regarding the impact of resection on functional outcomes [2,4,9,12,24,25,26,27,28,30,31,32,35]. This is critical, as improvement of functional status can open avenues for aggressive adjuvant therapies and clinical trial participation, which ultimately may improve survival [32]. Conceptually, the use of surgery as a bridge to further therapy has been demonstrated in initial brain metastasis resection, and there is data suggesting this may hold true for recurrent lesions [36]. Resection of recurrent metastases in a series of 80 patients led to a median 10-point increase in Karnofsky Performance Status (KPS) and overall survival of 11.1 months [9]. Notably, these authors reported that post-operative clinical status was the only independent predictor of post-operative survival in their multivariable analysis. However, they did caution about the high complication rate (~25%) in this patient population, 35% of which were neurological in nature. The reason for this is multifactorial but likely due to the complexity of the patient population, the elevated risk of a redo craniotomy and its associated challenges. Further, it is well established in multiple series that post-operative performance status significantly predicts survival [2,9,37]. Schackert et al. [4] found an increased risk of post-operative neurologic deterioration in patients with a pre-operative KPS < 70 compared to those with KPS > 70.

In addition to the impact on functional status, the oncological role of cytoreduction in recurrent brain metastasis has more recently been highlighted in the literature. This is a salient point, as many of these patients are in precarious oncological positions, including issues with systemic disease control and potentially more than one recurrent intracranial lesion to be addressed. Lin et al. [37] examined the role of surgery for recurrent brain metastases in a cohort of 219 patients (including patients with multiple recurrent lesions). Of the patients who underwent resection (n = 95), complete cytoreduction was accomplished in only 25%. As expected, patients with a lower number of lesions achieved higher levels of cytoreduction; specifically, complete cytoreduction was achieved in 87%, 11% and 2% of patients with one, two, and three brain metastases, respectively. No patient with greater than three metastases achieved complete cytoreduction of tumor burden. However, overall, they found that patients who underwent resection were afforded longer survival times compared to non-operative patients. Furthermore, the post-operative residual tumor burden significantly impacted patient survival, with a suggested cutoff for residual tumor volume of 0.12 cm^3^. Interestingly, residual tumor remained impactful in patients with uncontrolled systemic disease. Conversely, Arita et al. found that extent of resection had no significant effect on overall survival in a study of 33 patients with recurrent brain metastases after radiotherapy [38]. However, applicability of these findings may be limited given the very small patient population. Further study in larger patient cohorts is needed to characterize the optimal surgical plan for patients with recurrent brain metastases.

**Figure 1:**
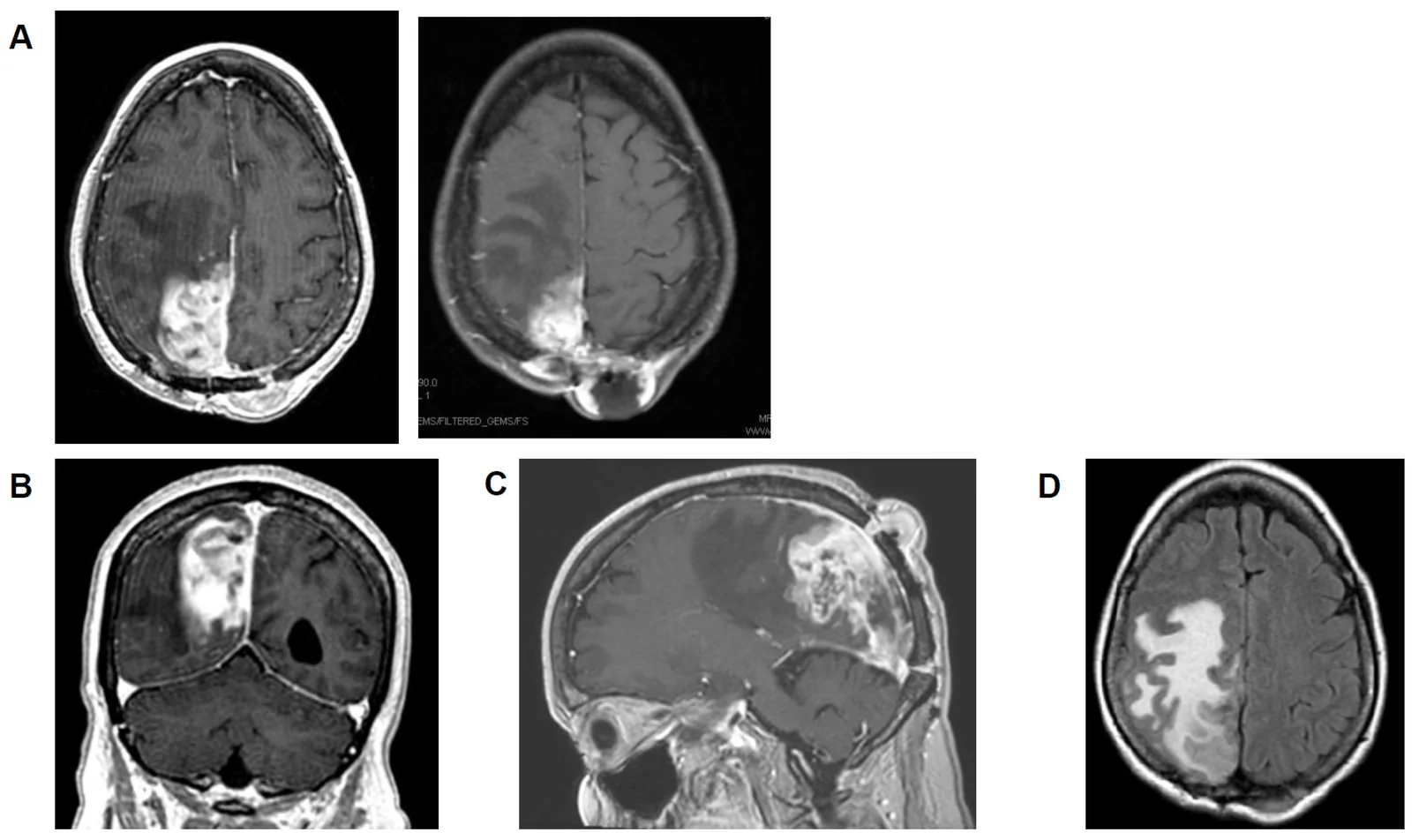
A 58 y/o female with BRAF mutant melanoma treated with two previous resections and two SRS treatments. She re-presented with headaches and left leg weakness. T1-weighted MRI with contrast (axial (**A**); coronal (**B**); sagittal (**C**)) showed a complex intracranial and extracranial re-recurrence involving the previous resection cavity, the adjacent calvarium and scalp. T2-weighted axial image (**D**) showed significant associated cerebral edema. She underwent re-resection followed by adjuvant WBRT.

As mentioned, decisions regarding candidacy for recurrent metastasis resection are complex. Patients are typically elderly, previously heavily treated for metastatic disease and may have medical co-morbidities to contend with. Patients with widespread systemic disease or immune compromise from systemic treatment may have compromised physiological reserve and are more prone to adverse outcomes. Further, patients require meaningful adjuvant options (either via radiation or systemic therapies) post-resection to reap survival benefits in terms of both intracranial and/or systemic disease control. As such, patients require careful selection to achieve maximal oncologic benefit (Figure 1). Hulsbergen et al. [2] reported one of the largest studies focused on surgical outcomes for recurrent metastasis and included 180 patients across multiple pathologies. These authors reported an overall survival post-resection of 13.8 months, with 57% of patients experiencing re-recurrence at 5.6 months (median). The authors noted that post-operative survival was significantly impacted by two factors, KPS and the presence of a targetable mutation. Specifically, HER2+ breast cancer and ALK/EGFR+ NSCLC patients with recurrence were afforded the longest survival times (>2 years). Interestingly, patients with recurrence from melanoma had the shortest post-operative survival time regardless of BRAF mutational status. Regarding the risk of local re-recurrence following resection, HER2+ breast cancer patients had the longest time to local recurrence following surgical resection compared to other included pathologies.

Overall, the data regarding the utility of reoperation for recurrent brain metastasis is growing. As patients live longer with advanced/metastatic disease, this patient population will likely grow along with the possibility of prospective and larger studies. Current studies of repeat metastasis resection are also frequently beset by methodological weaknesses. Patients undergo a wide variety of adjuvant treatments after their initial treatment and following resection of recurrent metastases, complicating any rigorous stratification across potential confounders; this problem is magnified by relatively small sample sizes. Retrospective, non-randomized designs also reduces the strength of these studies. However, available data does suggest that surgery does provide functional and oncologic benefit to carefully selected patients with accessible lesions and appropriate risk profiles.

## 4. Laser Interstitial Thermal Therapy

Laser interstitial thermal therapy (LITT) is a minimally invasive option for recurrent brain metastases that involves a small incision and burr hole with stereotactic placement of a laser probe for heat application [39]. Pathologic tissue is thought to have a higher absorption coefficient than healthy tissue, which facilitates safe and effective ablation [38,39]. Patients who are poor surgical candidates, have limited disease, small tumor size or those with difficult-to-reach/deep lesions may benefit from LITT [40]. LITT also has the advantage of providing access to tissue to distinguish between radiation necrosis and recurrent tumor, and LITT may be used to treat both [39]. However, patients with large or irregularly shaped lesions are at higher risk of recurrence following LITT [41]. Furthermore, blood–brain barrier disruption due to thermal injury may worsen tumor-associated edema and require prolonged steroid use [42]. Data regarding the role of LITT for recurrent metastases are mostly limited to small case series, though outcomes are promising. In a series of 12 patients with recurrent metastases who underwent LITT, median progression-free and overall survival were 7 and 25 months, respectively [43]. One patient in this series died due to cavity hemorrhage [43]. Salehi et al. [44] evaluated LITT in a cohort of 25 patients with brain metastases, 23 of which were recurrent. Median progression-free and overall survival were 6.3 and 13.3 months, respectively [44]. Kaye et al. [40] evaluated LITT in 70 patients with in-field metastatic recurrence following radiosurgery and reported that LITT was able to stabilize recurrent CNS disease in two-thirds of patients. Hong et al. [45] compared the efficacy of LITT to that of surgical resection in recurrent brain metastases following SRS in 42 patients and found that overall survival and progression-free survival were similar. Furthermore, Grabowski et al. [46] found that a combination of LITT and SRS led to significantly improved time to progression in 55 patients with recurrence following primary SRS compared to LITT or repeat SRS alone. Overall, LITT has shown promise and may be an option for patients with controlled systemic disease and suboptimal candidacy for open resection. However, larger studies comparing LITT to other modalities for management of recurrent disease are needed. Even though it is out of the scope of this review focused on tumor recurrence specifically, it is important to note that LITT is also a notable treatment option for radiation necrosis.

## 5. Intracavitary Brachytherapy

As a surgical resection adjunct, patients with recurrent brain metastases who are candidates for open surgery may also be evaluated for intracavitary brachytherapy implantation. Following resection for recurrent tumor, salvage radiation has been shown to be a viable adjuvant option [47]. Wilcox et al. [48] compared the benefit of adjuvant re-irradiation in 135 previously irradiated patients undergoing salvage resection of brain metastases and observed 6-month local recurrence rates of approximately 36% in patients who did not receive post-operative radiation and 18% in patients who did, though this difference was not statistically significant on multivariate analysis. A significant increase in 12-month rates of radiation necrosis was seen in patients undergoing re-irradiation (13.4% vs. 3.5%). [48] Repeat/salvage radiotherapy for recurrence confers an oncologic benefit, but this must be balanced against the compounded risk of radiation necrosis and other toxicities [48]. Radiation necrosis may significantly complicate or interrupt treatment, as it may require treatment with steroids, thus limiting the efficacy of immunotherapy or requiring delays in systemic treatment [49]. Whole-brain radiation therapy is considered a less favorable option due to substantial cognitive toxicity [50,51].

Intracavitary brachytherapy is a surgical treatment option for recurrent metastasis. At the time of surgery, radioactive seeds are placed directly into the cavity, allowing for immediate, continuous delivery of radiation dose, precluding microscopic tumor cell re-population/spread. Early trials of brachytherapy evaluated Iodine 125 seed implantation into the resection cavity [52,53,54]; however, more recent studies have focused on Cesium-131 (Cs-131) isotope brachytherapy [52,55,56,57,58,59,60,61,62,63,64,65,66,67,68]. This approach may have several advantages over conventional adjuvant radiotherapy. Firstly, the logistics are favorable, as radiation is delivered at the time of surgery, preventing missed treatments due to loss to follow-up or post-operative issues that may delay the timing of delivery of adjuvant therapy [63]. Additionally, intracavitary brachytherapy allows for a high degree of conformality, especially reducing post-operative surgical cavity dynamics that may make radiation planning more challenging [63]. Homogenous dose delivery can be accomplished even with an irregular surgical cavity, and dosimetry studies indicate that brachytherapy may allow for delivery of higher radiation doses with compared with external beam radiation methods. Further, by placing the radiation sources directly in the resection cavity, brachytherapy delivers high doses to the tumor bed while minimizing exposure to surrounding healthy brain tissue and achieves its peak dose at the periphery of the resection cavity, where tumor cells are more susceptible to irradiation [62]. This results in an overall low observed rate of radiation necrosis in modern series, ranging from 0 to 14% [62].

Regarding local control, intracavitary brachytherapy has shown oncological outcomes in several studies focused on recurrent brain metastasis [52,56,59,63]. One brachytherapy delivery option discussed in the literature is a collagen matrix (tile) impregnated with cesium 131 seeds that locally irradiate the surgical bed and a small margin of surrounding tissue [60]. A series of ten patients undergoing surgery and cesium-131 tile placement for recurrent brain metastases reported a local control rate of 100% at 18 months [63]. In another small series of 13 patients, Wernicke et al. [56] observed 12-month local control of 83.3% and median survival of 7 months following re-resection and intracavitary cesium 131 brachytherapy. A recent study by Beckham et al. reported the largest single-institution experience with cesium 131 brachytherapy for recurrent brain metastasis undergoing repeated resection and reported a cumulative incidence of local failure rate of 13% at 1 year and a low rate of radiation necrosis [61,64,69].

Overall, intracavitary brachytherapy appears to be a viable treatment option for patients with recurrent brain metastases. It is important to consider that it requires open surgical resection, so all patients may not be appropriate candidates. Furthermore, availability of this specialized modality may be limited, and it requires a multi-disciplinary team (surgeon, radiation oncology, radiation physicist, etc.) to execute. However, intracavitary therapy has demonstrated favorable local control rates with a low risk of radiation necrosis. In addition to producing favorable oncologic outcomes, Cesium 131 has also been shown to be cost effective in comparison to radiosurgery and to lead to improvements in neurocognitive function [57,58]. However, this strategy still requires larger patient series with longer follow-up times.

## 6. Repeat SRS

Recurrent brain metastases may be treated with an additional course of SRS. Historically, WBRT was the preferred salvage option due to its ability to address widespread intracranial disease. However, growing recognition of its associated neurotoxicity has shifted clinical preference toward more focal modalities like SRS [70]. The NCCN guidelines for the treatment of recurrent brain metastases indicate that reirradiation of metastases is a valid option once radiation necrosis (RN) has been ruled out. Furthermore, factors such as smaller lesion size and an interval of more than 6 months between SRS treatments have been associated with better outcomes [71,72]. Reirradiation via SRS carries its own risks, most notably an increased likelihood of RN. One study reported adverse radiation effects in as many as 30% of patients after repeat SRS, though this figure includes asymptomatic changes detected on imaging alone [72]. Multiple systematic reviews have estimated cumulative RN rates of 13–16% following repeat SRS [23,73,74].

The risk factors predisposing patients to RN in this setting remain incompletely characterized. Lucia et al. identified tumor volume and radiation dose as significant predictors, whereas Loi et al. and Singh et al. did not find these factors to be significant [73,74]. Loi et al. additionally reported an association between age at the time of retreatment and RN development, a finding that has not been replicated in other series [73]. Smaller studies have suggested additional potential risk factors, including the overlap in irradiated brain volumes between treatment courses and prior WBRT exposure, though these have not been validated in larger cohorts [75,76]. Fractionated SRS, rather than single-fraction delivery, may help reduce the risk of developing RN [76].

Despite these risks, repeat SRS has demonstrated promising oncologic outcomes across multiple systematic reviews. Local control rates after repeat SRS are estimated at 88.4–93% at 6 months, 69–76% at 1 year, and 54.2% at 2 years [23,73,74]. Factors associated with inferior local control across studies include melanoma primary tumor histology, prior WBRT, larger tumor volume, lower prescription dose, poorer KPS, and uncontrolled extracranial disease [23,73,74]. Singh et al. further quantified the dose–volume relationship, finding that each 1 Gy increase in marginal prescription dose was associated with a 5% improvement in local control, and each 1 cc increase in gross tumor volume at the first SRS was associated with a 5% reduction in local control; however, neither metric reached statistical significance (*p* = 0.14 and *p* = 0.11, respectively) [74].

Median overall survival following salvage SRS has been reported at approximately 14 months from retreatment, with 1-year survival rates of 49.7–54.2% [23,73,74]. McKay et al. identified a lower retreatment dose as a predictor of poorer survival outcomes [77]. For context, local control after primary SRS for brain metastases has been reported at approximately 93% at 6 months and 86% at 1 year, with a 1-year survival of 60%, suggesting that repeat SRS achieves broadly comparable efficacy to first-line treatment [78]. Furthermore, Kotecha et al. studied patients receiving three or more courses of SRS and found a 1-year overall survival rate of 71%, demonstrating the feasibility of multiple repeat treatments [79]. The modestly lower outcome rates observed in the repeat SRS setting are expected given the inherently more challenging biology and treatment history of recurrent disease. Outcomes may be further complicated by the concurrent development of RN, which can be difficult to distinguish radiographically from true progression and may confound both local control assessments and clinical decision-making [73].

## 7. Adjuvant Therapy Post-Salvage Resection

Although salvage resection alone can be an effective option for recurrent brain metastases, adding adjuvant treatment may provide further benefit. Adjuvant treatment options include SRS, WBRT, and intracavitary brachytherapy, all of which aim to prevent further disease progression/re-recurrence and prolong survival. Evidence for adjuvant treatment after salvage resection varies widely. Several studies have found that adjuvant radiation, including WBRT and SRS, has no significant effect on patient survival or disease progression [4,31,32,48,80]. A meta-analysis by Basaran et al. similarly failed to find a significant survival benefit from adjuvant radiation after repeat resection [81].

However, other studies have shown a significant benefit. Cummins et al. found that both brachytherapy and SRS significantly improved local control when salvage surgery was performed at least 4.5 months after initial SRS [80]. It should be noted, though, that the primary treatment prior to salvage resection in this study was SRS [80]. Wilcox et al. further supported a possible benefit for local control, reporting a 12-month local recurrence rate of 28.8% with adjuvant radiotherapy compared to 43.9% without, though this difference was not statistically significant (*p* = 0.07) [48]. Radiation modality (SRS, WBRT, or partial brain radiotherapy) did not affect local control, and overall survival was not significantly improved [48]. Adverse radiation effects must also be considered, as adjuvant radiation carried a significantly higher risk of radiation necrosis, though most cases were asymptomatic [48]. In Wu et al., brachytherapy also improved local control and extended overall survival [69]. However, this study was not restricted to patients who had undergone salvage resection, as 5 of 14 patients had undergone only one surgery in their prior disease course. Although evidence for post-salvage resection adjuvant therapy is limited, the studies discussed here point to a potential benefit. Buszek et al. [47] reached a similar conclusion: among patients who underwent gross total resection, presence of tumor on pathology was the only significant predictor of local treatment failure, and 47 of 118 such patients who did not receive adjuvant therapy developed local recurrence at a median of 4 months, demonstrating the limited efficacy of observation alone [47]. From this limited pool, brachytherapy appears to be a promising adjuvant option after salvage resection, but there is insufficient evidence to rule out radiotherapy or systemic therapy, and further studies are needed to investigate these options.

## 8. Management of Multiple Recurrent Tumors

As the survival of cancer patients improves, many are experiencing second and third recurrences of brain metastases. Additionally, the rate of recurrence after treatment of first-time recurrence is considerable, ranging from 37 to 43% [44]. Several authors have attempted to identify risk factors for multiple recurrences. Extent of resection was the only risk factor identified in a series of 37 patients treated with repeat craniotomy for recurrence [12]. A larger series of 170 patients with second recurrence of brain metastases identified extent of resection and infratentorial location as predictors of further recurrence [32]. As the management of metastases at first recurrence is not standardized, the management of second recurrences and beyond is also highly individualized. In a series of 26 patients with second-time recurrence after two prior surgeries, Bindal et al. [26] found a significant survival advantage of surgery (8.6 vs. 2.8 months). Kamp et al. [12] also observed a survival advantage (12.9 vs. 8.4 months). Few data exist on adjuvant treatments for multiply recurrent tumors, given the limited number of patients who survive to this stage.

## 9. Further Directions

Studies of recurrent brain metastases have several general limitations that hinder their generalizability and are likely to explain the wide individual variation in practice. Firstly, cancer patients vary widely in clinical status from asymptomatic to chronically systemically ill and thus have varying tolerance to cancer therapy [9]. Additionally, the treatment regimens previously undergone by patients in these studies vary widely, limiting direct comparison [63]. Brain metastases behave differently by histology in response to systemic treatment and radiation. Furthermore, as the systemic disease of some patients invariably progresses, these patients are often lost to follow-up, while patients who are clinically stable are more reliably included and thereby possibly lead to survival bias [11].

## 10. Conclusions

The management of recurrent brain tumors is highly individualized, though many options for treatment exist depending on the clinical status of the patient. From a surgical perspective, re-resection is an option for patients with surgically accessible metastases and good performance status. Not only does surgery provide oncological and functional benefits in selected patients, but it also provides diagnostic confirmation for previously treated lesions, which can be a challenge [82,83]. Despite initial concerns regarding risks of radiation necrosis, re-irradiation after both whole-brain radiotherapy and SRS is a viable option with good rates of local control and an acceptable toxicity profile. However, intracavitary brachytherapy is a promising alternative to adjuvant stereotactic radiation with logistical and radiobiological advantages. Patients with deep-seated lesions or poor candidates for traditional, definitive resection may be candidates for LITT, which offers minimally invasive cytoreduction. Despite limited data from small series, patients with multiple recurrences may still be candidates for further treatment depending on their performance status.

## Figures and Tables

**Table 1:** Studies investigating resection of recurrent brain metastases following resection of primary metastasis.

Study Author and Year	N	Primary	Extent of Resection	Complication Rate	Peri-Op Mortality	Median Time to Recurrence	Recurrence	Number of Patients Developed Second Recurrence	Median Survival Time Post-Second Craniotomy
Sundaresan et al. [24], 1988	21	Multiple	GTR (18), STR (3)	23.8%	0%	3–30 months ^#^	Local (14), Distant (7)	5	9 months
Arbit et al. [25], 1995	32 (109 *)	NSCLC (32)	N/A	31%	0%	5 months **	Local (53), Distant (16), Both (15), Multiple new tumors (16), Meningeal Carcinomatosis (9) *	8	10 months
Bindal et al. [26], 1995	48	Multiple	N/A	10.42%	0%	6.7 months	Local (30), Distant (16), Both (2)	26 (Local (18), Distant (3), Both (5))	11.5 months
Zacest et at. [27], 2002	24 (147 *)	Melanoma (24)	GTR (125), STR (19), Biopsy Only (3) *	19.05%	3.40% *	8 months **	Local (14), Distant (10)	3	15 months
Koutras et al. [28], 2003	6 (32 *)	NSCLC (6)	GTR (31), STR (1) *	N/A	0%	N/A	N/A	1	16.5 months ***
Al-Zabin et al. [29], 2010	25	Lung (25)	GTR (29)	0%	0%	7 months	Local (21), Distant (4)	N/A	8.3 months
Miller et al. [30], 2013	6 (34 *)	Melanoma (6)	N/A	N/A	N/A	5.5 months *^,++^	Local (7), Distant (11) *	N/A	6 months ^+^
Schackert et al. [4], 2013	67	Multiple	GTR (39), STR (28)	N/A	N/A	6 months	Local (35), Distant (13), Both (19)	21 (16 reoperations)	7.5 months
Kennion et al. [31], 2017	29	Multiple	N/A	6.90%	6.8%	7 months	Local (26), Distant (3)	14 (5 reoperations)	7.6 months
Kamp et al. [12], 2018	36 (37 lesions)	Multiple	GTR (15), STR (14), Questionable (8)	0%	5.56%	9.3 months **^,++^	Local (37)	24 (14 local, 10 distant)	12.9 months
Hulsbergen et al. [2], 2021	63 (180 *)	Multiple	GTR (102), STR (75), Undetermined (3) *	N/A	1.7% *	5.6 months *	Local (16), Distant (47)	28	14.7 months
Hessler et al. [9], 2022	44 (107 *)	Multiple	GTR (78), STR (29) *	26.2% *	2.80% * (only 2 from surgical complications) *	N/A	N/A	51 *	11.1 months *
Tewarie et al. [32], 2022 ^+++^	161 (170 lesions)	Multiple	STR (85), GTR (85)	43.5%	N/A	N/A	Local (170)	74	N/A
Goldberg et al. [33], 2024	95	N/A	N/A	N/A	N/A	N/A	N/A	N/A	6 months (65 and over), 8 months (<65)
Telera et al. [34], 2024	37	Multiple	GTR (34), STR (3)	8.1%	2.7%	8 months **	Local (37)	N/A	7 months
Wasilewski et al. [35], 2025	60	Multiple	GTR (29), STR (8), Unknown (23)	15%	N/A	7.7 months	Local (41), Distant (19)	N/A	11 months

STR = subtotal resection, GTR = gross total resection, DVT = deep vein thrombosis, NSCLC = non-small cell lung cancer, SCLC = small cell lung cancer, RCC = renal cell carcinoma, CUP = cancer of unknown primary; * data based on full study, including patients with either non-surgical primary or recurrence treatment; ** reported time from first to second craniotomy, rather than first craniotomy to diagnosis of recurrence; *** post-first craniotomy, rather than post-second craniotomy; + includes 5 patients who received radiosurgery for recurrence; ++ data reported as mean rather than median; +++ data reported in units of lesions, not patients; # data reported as range rather than median.

## Data Availability

No new data were created or analyzed in this study. Data sharing is not applicable to this article.

## Abbreviations

The following abbreviations are used in this manuscript:

CNS	Central nervous system
SRS	Stereotactic Radiosurgery
WBRT	Whole-brain radiation therapy
KPS	Karnofsky performance status
LITT	Laser interstitial thermal therapy

## References

[1] Sankey E.W., Tsvankin V., Grabowski M.M., Nayar G., Batich K.A., Risman A., Champion C.D., Salama A.K.S., Goodwin C.R., Fecci P.E. (2019). Operative and peri-operative considerations in the management of brain metastasis. Cancer Med..

[2] Hulsbergen A.F.C., Abunimer A.M., Ida F., Kavouridis V.K., Cho L.D., Tewarie I.A., Mekary R.A., Schucht P., Phillips J.G., Verhoeff J.J.C. (2021). Neurosurgical resection for locally recurrent brain metastasis. Neuro-Oncology.

[3] Patel A.J., Suki D., Hatiboglu M.A., Abouassi H., Shi W., Wildrick D.M., Lang F.F., Sawaya R. (2010). Factors influencing the risk of local recurrence after resection of a single brain metastasis. J. Neurosurg..

[4] Schackert G., Schmiedel K., Lindner C., Leimert M., Kirsch M. (2013). Surgery of recurrent brain metastases: Retrospective analysis of 67 patients. Acta Neurochir..

[5] Schnurman Z., Mashiach E., Link K.E., Donahue B., Sulman E., Silverman J., Golfinos J.G., Oermann E.K., Kondziolka D. (2023). Causes of Death in Patients with Brain Metastases. Neurosurgery.

[6] Vogelbaum M.A., Brown P.D., Messersmith H., Brastianos P.K., Burri S., Cahill D., Dunn I.F., Gaspar L.E., Gatson N.T.N., Gondi V. (2022). Treatment for Brain Metastases: ASCO-SNO-ASTRO Guideline. J. Clin. Oncol..

[7] Ammirati M., Cobbs C.S., Linskey M.E., Paleologos N.A., Ryken T.C., Burri S.H., Asher A.L., Loeffler J.S., Robinson P.D., Andrews D.W. (2010). The role of retreatment in the management of recurrent/progressive brain metastases: A systematic review and evidence-based clinical practice guideline. J. Neuro-Oncol..

[8] Chidambaram S., Pannullo S.C., Schwartz T.H., Wernicke A.G. (2019). Reirradiation of Recurrent Brain Metastases: Where Do We Stand?. World Neurosurg..

[9] Heßler N., Jünger S.T., Meissner A.-K., Kocher M., Goldbrunner R., Grau S. (2022). Recurrent brain metastases: The role of resection of in a comprehensive multidisciplinary treatment setting. BMC Cancer.

[10] Munoz-Bendix C., Rapp M., Mijderwijk H.-J., von Sass C., Dibué-Adjei M., Cornelius J.F., Steiger H.-J., Turowski B., Sabel M., Kamp M.A. (2019). Risk factors for in-brain local progression in elderly patients after resection of cerebral metastases. Sci. Rep..

[11] Aftahy A.K., Barz M., Lange N., Baumgart L., Thunstedt C., Eller M.A., Wiestler B., Bernhardt D., Combs S.E., Jost P.J. (2022). The Impact of Postoperative Tumor Burden on Patients with Brain Metastases. Front. Oncol..

[12] Kamp M.A., Fischer I., Dibué-Adjei M., Munoz-Bendix C., Cornelius J.-F., Steiger H.-J., Slotty P.J., Turowski B., Rapp M., Sabel M. (2018). Predictors for a further local in-brain progression after re-craniotomy of locally recurrent cerebral metastases. Neurosurg. Rev..

[13] She C., Wang R., Lu C., Sun Z., Li P., Yin Q., Liu Q., Wang P., Li W. (2019). Prognostic factors and outcome of surgically treated patients with brain metastases of non-small cell lung cancer. Thorac. Cancer.

[14] McHugh F.A., Kow C.Y., Falkov A., Heppner P., Law A., Bok A., Schweder P. (2020). Metastatic melanoma: Surgical treatment of brain metastases—Analysis of 110 patients. J. Clin. Neurosci..

[15] Winther R.R., Hjermstad M.J., Skovlund E., Aass N., Helseth E., Kaasa S., Yri O.E., Vik-Mo E.O. (2022). Surgery for brain metastases-impact of the extent of resection. Acta Neurochir..

[16] Olesrud I.C., Schulz M.K., Marcovic L., Kristensen B.W., Pedersen C.B., Kristiansen C., Poulsen F.R. (2019). Early postoperative MRI after resection of brain metastases-complete tumour resection associated with prolonged survival. Acta Neurochir..

[17] Hulsbergen A.F.C., Siddi F., Cerecedo C., Robertson F.C., Lo Y.-T., Kavouridis V., Ehret F., Jessurun C.A.C., Tewarie I.R., Phillips J.G. (2026). Impact of Extent of Resection on Survival in Brain Metastasis: An Analysis of 867 Patients. Neurosurgery.

[18] O’Brien D.A.R., Kaye S.M., Poppas P.J., Mahase S.S., An A., Christos P.J., Liechty B., Pisapia D., Ramakrishna R., Wernicke A.G. (2021). Time to administration of stereotactic radiosurgery to the cavity after surgery for brain metastases: A real-world analysis. J. Neurosurg..

[19] O’Brien D.A.R., Poppas P., Kaye S.M., Mahase S.S., An A., Christos P.J., Liechty B., Pisapia D., Ramakrishna R., Wernicke A.G. (2021). Timing of Adjuvant Fractionated Stereotactic Radiosurgery Affects Local Control of Resected Brain Metastases. Pract. Radiat. Oncol..

[20] Lamba N., Cagney D.N., Catalano P.J., Kim D., Elhalawani H., Haas-Kogan D.A., Wen P.Y., Wagle N., Aizer A.A. (2023). A genomic score to predict local control among patients with brain metastases managed with radiation. Neuro-Oncology.

[21] Rashid N.S., Lamba N., Catalano P.J., Elhalawani H., Tanguturi S.K., Rahman R., Haas-Kogan D.A., Wen P.Y., Aizer A.A. (2025). Impact of brain metastasis size at the time of radiotherapy on local control and radiation necrosis. J. Neuro-Oncol..

[22] Brigell R.H., Cagney D.N., Martin A.M., Besse L.A., Catalano P.J., Lee E.Q., Wen P.Y., Brown P.D., Phillips J.G., Pashtan I.M. (2019). Local control after brain-directed radiation in patients with cystic versus solid brain metastases. J. Neuro-Oncol..

[23] Lucia F., Touati R., Crainic N., Dissaux G., Pradier O., Bourbonne V., Schick U. (2021). Efficacy and Safety of a Second Course of Stereotactic Radiation Therapy for Locally Recurrent Brain Metastases: A Systematic Review. Cancers.

[24] Sundaresan N., Sachdev V.P., DiGiacinto G.V., Hughes J.E. (1988). Reoperation for brain metastases. J. Clin. Oncol..

[25] Arbit E., Wroński M., Burt M., Galicich J.H. (1995). The treatment of patients with recurrent brain metastases. A retrospective analysis of 109 patients with nonsmall cell lung cancer. Cancer.

[26] Bindal R.K., Sawaya R., Leavens M.E., Hess K.R., Taylor S.H. (1995). Reoperation for recurrent metastatic brain tumors. J. Neurosurg..

[27] Zacest A.C., Besser M., Stevens G., Thompson J.F., McCarthy W.H., Culjak G. (2002). Surgical management of cerebral metastases from melanoma: Outcome in 147 patients treated at a single institution over two decades. J. Neurosurg..

[28] Koutras A.K., Marangos M., Kourelis T., Partheni M., Dougenis D., Iconomou G., Vagenakis A.G., Kalofonos H.P. (2003). Surgical management of cerebral metastases from non-small cell lung cancer. Tumori J..

[29] Al-Zabin M., Ullrich W.O., Brawanski A., Proescholdt M.A. (2010). Recurrent brain metastases from lung cancer: The impact of reoperation. Acta Neurochir..

[30] Miller D., Zappala V., El Hindy N., Livingstone E., Schadendorf D., Sure U., Sandalcioglu I.E. (2013). Intracerebral metastases of malignant melanoma and their recurrences—A clinical analysis. Clin. Neurol. Neurosurg..

[31] Kennion O., Holliman D. (2017). Outcome after craniotomy for recurrent cranial metastases. Br. J. Neurosurg..

[32] Tewarie I.A., Hulsbergen A.F.C., Jessurun C.A.C., Rendon L.F., Mekary R.A., Smith T.R., Broekman M.L.D. (2022). Risk Factors of Second Local Recurrence in Surgically Treated Recurrent Brain Metastases: An Exploratory Analysis. World Neurosurg..

[33] Goldberg M., Heinrich V., Altawalbeh G., Negwer C., Wagner A., Gempt J., Meyer B., Aftahy A.K. (2024). The Role of Repeated Surgical Resections for Recurrent Brain Metastases in Older Population. Medicina.

[34] Telera S., Tosatto L., Colasanti R., Pace A., Villani V., Rasile F., Lecce M., Crispo F., Marucci L., Farneti A. (2024). The role of surgery in recurrent local cerebral metastases: A multi-institutional retrospective analysis. Neurosurg. Rev..

[35] Wasilewski D., Shaked Z., Fuchs A., Roohani S., Xu R., Schlaak M., Frost N., Misch M., Capper D., Kaul D. (2025). Re-resection of brain metastases—Outcomes of an institutional cohort study and literature review. BMC Cancer.

[36] Schödel P., Jünger S.T., Wittersheim M., Reinhardt H.C., Schmidt N.-O., Goldbrunner R., Proescholdt M., Grau S. (2020). Surgical resection of symptomatic brain metastases improves the clinical status and facilitates further treatment. Cancer Med..

[37] Lin J., Kaiser Y., Wiestler B., Bernhardt D., Combs S.E., Delbridge C., Meyer B., Gempt J., Aftahy A.K. (2023). Cytoreduction of Residual Tumor Burden Is Decisive for Prolonged Survival in Patients with Recurrent Brain Metastases—Retrospective Analysis of 219 Patients. Cancers.

[38] Arita H., Ikawa T., Kanayama N., Morimoto M., Umehara T., Yoshizawa H., Kodama Y., Okita Y., Kinoshita M., Konishi K. (2025). Prognostic factors after salvage resection for local progression of brain metastases after radiotherapy. Acta Neurochir..

[39] Damante M.A., Wang J.L., Elder J.B. (2022). Surgical Management of Recurrent Brain Metastasis: A Systematic Review of Laser Interstitial Thermal Therapy. Cancers.

[40] Kaye J., Patel N.V., Danish S.F. (2020). Laser interstitial thermal therapy for in-field recurrence of brain metastasis after stereotactic radiosurgery: Does treatment with LITT prevent a neurologic death?. Clin. Exp. Metastasis.

[41] Sanvito F., Telesca D., Cho N.S., Sayari J.T., Nagaraj R., Raymond C., Rana S., Patel K., Mozaffari K., Unterberger A.A. (2024). Small pretreatment lesion size and high sphericity as favorable prognostic factors after laser interstitial thermal therapy in brain metastases. J. Neurosurg..

[42] McGrath K., Frain M., Hey G., Rahman M. (2025). Complications following laser interstitial thermal therapy: A review. Neurochirurgie.

[43] Dabecco R., Gigliotti M.J., Mao G., Myers D., Xu L., Lee P., Ranjan T., Aziz K., Yu A. (2024). Laser interstitial thermal therapy (LITT) for intracranial lesions: A single-institutional series, outcomes, and review of the literature. Br. J. Neurosurg..

[44] Salehi A., Kamath A.A., Leuthardt E.C., Kim A.H. (2018). Management of Intracranial Metastatic Disease with Laser Interstitial Thermal Therapy. Front. Oncol..

[45] Hong C.S., Deng D., Vera A., Chiang V.L. (2019). Laser-interstitial thermal therapy compared to craniotomy for treatment of radiation necrosis or recurrent tumor in brain metastases failing radiosurgery. J. Neuro-Oncol..

[46] Grabowski M.M., Srinivasan E.S., Vaios E.J., Sankey E.W., Otvos B., Krivosheya D., Scott A., Olufawo M., Ma J., Fomchenko E.I. (2022). Combination laser interstitial thermal therapy plus stereotactic radiotherapy increases time to progression for biopsy-proven recurrent brain metastases. Neuro-Oncol. Adv..

[47] Buszek S.M., Tran B., Long J.P., Luo D., Suki D., Li J., Ferguson S., Chung C. (2023). Postoperative Management of Recurrence After Radiosurgery and Surgical Resection for Brain Metastases and Predicting Benefit from Adjuvant Radiation. Pract. Radiat. Oncol..

[48] Wilcox J.A., Brown S., Reiner A.S., Young R.J., Chen J., Bale T.A., Rosenblum M.K., Newman W.C., Brennan C.W., Tabar V. (2021). Salvage resection of recurrent previously irradiated brain metastases: Tumor control and radiation necrosis dependency on adjuvant re-irradiation. J. Neuro-Oncol..

[49] Kowalchuk R.O., Niranjan A., Lee C.-C., Yang H.-C., Liscak R., Guseynova K., Tripathi M., Kumar N., Peker S., Samanci Y. (2022). Reirradiation with Stereotactic Radiosurgery After Local or Marginal Recurrence of Brain Metastases from Previous Radiosurgery. Int. J. Radiat. Oncol. Biol. Phys..

[50] Maranzano E., Terenzi S., Anselmo P., Casale M., Arcidiacono F., Loreti F., Di Marzo A., Draghini L., Italiani M., Trippa F. (2019). A prospective phase II trial on reirradiation of brain metastases with radiosurgery. Clin. Transl. Radiat. Oncol..

[51] Chang C.H., Horton J., Schoenfeld D., Salazer O., Perez-Tamayo R., Kramer S., Weinstein A., Nelson J.S., Tsukada Y. (1983). Comparison of postoperative radiotherapy and combined postoperative radiotherapy and chemotherapy in the multidisciplinary management of malignant gliomas. A joint Radiation Therapy Oncology Group and Eastern Cooperative Oncology Group study. Cancer.

[52] Palmisciano P., Haider A.S., Balasubramanian K., Boockvar J.A., Schwartz T.H., D’Amico R.S., Gabriella Wernicke A. (2023). Cesium-131 brachytherapy for the treatment of brain metastases: Current status and future perspectives. J. Clin. Neurosci..

[53] Raleigh D.R., Solomon D.A., Lloyd S.A., Lazar A., Garcia M.A., Sneed P.K., Clarke J.L., McDermott M.W., Berger M.S., Tihan T. (2017). Histopathologic review of pineal parenchymal tumors identifies novel morphologic subtypes and prognostic factors for outcome. Neuro-Oncology.

[54] Romagna A., Schwartz C., Egensperger R., Watson J., Tonn J.-C., Belka C., Kreth F.-W., Nachbichler S.B. (2016). Iodine-125 brachytherapy as upfront and salvage treatment for brain metastases: A comparative analysis. Strahlenther. Onkol..

[55] Wernicke A.G., Yondorf M.Z., Peng L., Trichter S., Nedialkova L., Sabbas A., Kulidzhanov F., Parashar B., Nori D., Clifford Chao K.S. (2014). Phase I/II study of resection and intraoperative cesium-131 radioisotope brachytherapy in patients with newly diagnosed brain metastases. J. Neurosurg..

[56] Wernicke A.G., Smith A.W., Taube S., Yondorf M.Z., Parashar B., Trichter S., Nedialkova L., Sabbas A., Christos P., Ramakrishna R. (2017). Cesium-131 brachytherapy for recurrent brain metastases: Durable salvage treatment for previously irradiated metastatic disease. J. Neurosurg..

[57] Wernicke A.G., Yondorf M.Z., Parashar B., Nori D., Clifford Chao K.S., Boockvar J.A., Pannullo S., Stieg P., Schwartz T.H. (2016). The cost-effectiveness of surgical resection and cesium-131 intraoperative brachytherapy versus surgical resection and stereotactic radiosurgery in the treatment of metastatic brain tumors. J. Neuro-Oncol..

[58] Pham A., Yondorf M.Z., Parashar B., Scheff R.J., Pannullo S.C., Ramakrishna R., Stieg P.E., Schwartz T.H., Wernicke A.G. (2016). Neurocognitive function and quality of life in patients with newly diagnosed brain metastasis after treatment with intra-operative cesium-131 brachytherapy: A prospective trial. J. Neuro-Oncol..

[59] Chen W.C., Lafreniere M., Phuong C., Liu S.J., Baal J.D., Lometti M., Morin O., Ziemer B., Vasudevan H.N., Lucas C.-H.G. (2022). Resection with intraoperative cesium-131 brachytherapy as salvage therapy for recurrent brain tumors. J. Neurosurg..

[60] Imber B.S., Young R.J., Beal K., Reiner A.S., Giantini-Larsen A.M., Krebs S., Yang J.T., Aramburu-Nunez D., Cohen G.N., Brennan C. (2022). Salvage resection plus cesium-131 brachytherapy durably controls post-SRS recurrent brain metastases. J. Neuro-Oncol..

[61] Beckham T.H., Cha E.E., Rooney M.K., Tom M.C., Perni S., McAleer M.F., Li J., Kudchadker R.J., Prajapati S., Zlateva Y. (2025). Cesium-131 collagen tile brachytherapy for salvage of recurrent intracranial metastases. J. Neuro-Oncol..

[62] Bander E.D., Knisely J.P.S., Schwartz T.H. (2022). Brachytherapy for central nervous system tumors. J. Neuro-Oncol..

[63] Kutuk T., Tolakanahalli R., Chaswal V., Yarlagadda S., Herrera R., Appel H., La Rosa A., Mishra V., Wieczorek D.J.J., McDermott M.W. (2023). Surgically targeted radiation therapy (STaRT) for recurrent brain metastases: Initial clinical experience. Brachytherapy.

[64] Yu K.K.H., Imber B.S., Moss N.S. (2021). Multimodality durable salvage of recurrent brain metastases refractory to LITT, SRS and immunotherapy with resection and cesium-131 brachytherapy: Case report and literature review. BMJ Case Rep..

[65] Dharnipragada R., Ferreira C., Shah R., Reynolds M., Dusenbery K., Chen C.C. (2023). GammaTile® (GT) as a brachytherapy platform for rapidly growing brain metastasis. Neuro-Oncol. Adv..

[66] Nakaji P., Smith K., Youssef E., Thomas T., Pinnaduwage D., Rogers L., Wallstrom G., Brachman D. (2020). Resection and Surgically Targeted Radiation Therapy for the Treatment of Larger Recurrent or Newly Diagnosed Brain Metastasis: Results from a Prospective Trial. Cureus.

[67] Warren K.T., Boucher A., Bray D.P., Dresser S., Zhong J., Shu H.-K., Olson J., Hoang K., Warren K.T., Boucher A.B. (2021). Surgical Outcomes of Novel Collagen Tile Cesium Brachytherapy for Recurrent Intracranial Tumors at a Tertiary Referral Center. Cureus.

[68] Arsenault T., Labak C.M., Chaung K., Muzic R.F., Kardan A., Sloan A., Choi S., Podder T., Ouyang Z. (2021). Treating Recurrent Brain Metastases Using GammaTile Brachytherapy: A Case Report and Dosimetric Modeling Method. Cureus.

[69] Wu K.C., Cantalino J.M., Dee E.C., Hsu L., Harris T.C., Rawal B., Juvekar P.R., Mooney M.A., Dunn I.F., Aizer A.A. (2022). Salvage brachytherapy for multiply recurrent metastatic brain tumors: A matched case analysis. Neuro-Oncol. Adv..

[70] Laabid K., Abahssain H., Audouard R.M., Khouchani M., Pasquier D. (2026). Salvage stereotactic radiotherapy for locally recurrent brain metastases: A systematic review. Radiat. Oncol..

[71] Nabors L.B., Portnow J., Ahluwalia M., Baehring J., Brem H., Brem S., Butowski N., Campian J.L., Clark S.W., Fabiano A.J. (2020). Central Nervous System Cancers, Version 3.2020, NCCN Clinical Practice Guidelines in Oncology. J. Natl. Compr. Cancer Netw..

[72] Sneed P.K., Chan J.W., Ma L., Braunstein S.E., Theodosopoulos P.V., Fogh S.E., Nakamura J.L., Boreta L., Raleigh D.R., Ziemer B.P. (2023). Adverse radiation effect and freedom from progression following repeat stereotactic radiosurgery for brain metastases. J. Neurosurg..

[73] Loi M., Caini S., Scoccianti S., Bonomo P., De Vries K., Francolini G., Simontacchi G., Greto D., Desideri I., Meattini I. (2020). Stereotactic reirradiation for local failure of brain metastases following previous radiosurgery: Systematic review and meta-analysis. Crit. Rev. Oncol. Hematol..

[74] Singh R., Didwania P., Lehrer E.J., Palmer J.D., Trifiletti D.M., Sheehan J.P. (2022). Repeat stereotactic radiosurgery for locally recurrent brain metastases previously treated with stereotactic radiosurgery: A systematic review and meta-analysis of efficacy and safety. J. Radiosurg. SBRT.

[75] Minniti G., Scaringi C., Paolini S., Lanzetta G., Romano A., Cicone F., Osti M., Enrici R.M., Esposito V. (2016). Single-Fraction Versus Multifraction (3 × 9 Gy) Stereotactic Radiosurgery for Large (>2 cm) Brain Metastases: A Comparative Analysis of Local Control and Risk of Radiation-Induced Brain Necrosis. Int. J. Radiat. Oncol. Biol. Phys..

[76] Yan M., Lee M., Myrehaug S., Tseng C.-L., Detsky J., Chen H., Das S., Yeboah C., Lipsman N., Costa L.D. (2023). Hypofractionated stereotactic radiosurgery (HSRS) as a salvage treatment for brain metastases failing prior stereotactic radiosurgery (SRS). J. Neuro-Oncol..

[77] McKay W.H., McTyre E.R., Okoukoni C., Alphonse-Sullivan N.K., Ruiz J., Munley M.T., Qasem S., Lo H.-W., Xing F., Laxton A.W. (2017). Repeat stereotactic radiosurgery as salvage therapy for locally recurrent brain metastases previously treated with radiosurgery. J. Neurosurg..

[78] Habibi Z., Ertiaei A., Nikdad M.S., Mirmohseni A.S., Afarideh M., Heidari V., Saberi H., Rezaei A.S., Nejat F. (2016). Predicting ventriculoperitoneal shunt infection in children with hydrocephalus using artificial neural network. Child’s Nerv. Syst..

[79] Kotecha R., Damico N., Miller J.A., Suh J.H., Murphy E.S., Reddy C.A., Barnett G.H., Vogelbaum M.A., Angelov L., Mohammadi A.M. (2017). Three or More Courses of Stereotactic Radiosurgery for Patients with Multiply Recurrent Brain Metastases. Neurosurgery.

[80] Cummins D.D., Morshed R.A., Chavez M.M., Avalos L.N., Sudhakar V., Chung J.E., Gallagher A., Saggi S., Daras M., Braunstein S. (2022). Salvage Surgery for Local Control of Brain Metastases After Previous Stereotactic Radiosurgery: A Single-Center Series. World Neurosurg..

[81] Basaran A.E., Fahsold L., Lordick F., Nicolay N.H., Güresir E., Wach J. (2025). Treatment modalities in recurrent brain metastases: A combined institutional and individual patient data meta-analysis of post-recurrence survival and local progression-free survival. J. Neuro-Oncol..

[82] Marchal J., Ybert L., Ducloie M., Geffrelot J., Faisant M., Braux G., Lesueur P., Goliot N., Pernin V., Gaberel T. (2026). The threefold value of surgery in previously irradiated brain metastases. J. Neuro-Oncol..

[83] Kotecha R., Chiang V., Tom M.C., Parent E., Nabavizadeh A., Siegel B.A., Huang J., Zan E., Peddi S., Brem S. (2026). 18F-Fluciclovine for Detection of Recurrent Brain Metastases After Radiation Therapy: Image Interpretation Criteria and Diagnostic Performance from the PURSUE Study. Int. J. Radiat. Oncol. Biol. Phys..

